# Effective dose and image optimisation of lateral lumbar spine radiography: a phantom study

**DOI:** 10.1186/s41747-019-0132-3

**Published:** 2020-02-13

**Authors:** Zer Hau Lai, Cláudia Sá dos Reis, Zhonghua Sun

**Affiliations:** 1 Discipline of Medical Radiation Sciences, School of Molecular and Life Sciences, Curtin University, GPO Box U1987, Perth, Western Australia 6845 Australia; 2 School of Health Sciences (HESAV), University of Applied Sciences and Arts Western Switzerland (HES-SO), Av. de Beaumont 21, 1011 Lausanne, Switzerland; 3 NOVA National School of Public Health, Public Health Research Centre, Universidade NOVA de Lisboa, Lisbon, Portugal

**Keywords:** Image quality, Lumbosacral region, Phantoms (imaging), Radiation dosage, Radiography

## Abstract

**Background:**

To investigate lateral lumbar spine radiography technical parameters for reduction of effective dose whilst maintaining image quality (IQ).

**Methods:**

Thirty-six radiograms of an anthropomorphic phantom were acquired using different exposure parameters: source-to-detector distance (SDD) (100, 130 or 150 cm), tube potential (75, 85 or 95 kVp), tube current × exposure time product (4.5, 9, 18 mAs) and additional copper (Cu) filter (no filter, 0.1-, 0.2-, or 0.3-mm thickness. IQ was assessed using an objective approach (contrast-to-noise-ratio [CNR] calculation and magnification measurement) and a perceptual approach (six observers); ED was estimated using the PCXMC 2.0 software. Descriptive statistics, paired *t* test, and intraclass correlation coefficient (ICC) were used.

**Results:**

The highest ED (0.022 mSv) was found with 100 cm SSD, 75 kVp, 18 mAs, and without Cu filter, whilst the highest CNR (7.23) was achieved at 130 cm SSD, 75 kVp, 18 mAs, and without Cu filter. The lowest ED and CNR were generated at 150 cm SDD, 95 kVp, 4.5 mAs, and 0.3-mm Cu filter. All observers identified the relevant anatomical structures on all images with the lowest ED and IQ. The intra-observer (0.61–0.79) and inter-observer (0.55–0.82) ICC ranged from moderate to excellent.

**Conclusion:**

All relevant anatomical structures were identified on the lateral lumbar spine radiographs despite using low-dose protocols. The lowest ED (0.002 mSv) was obtained with 150 cm SDD, 95 kVp, 4.5 mAs, and 0.3-mm Cu filter. Further technical and clinical studies are needed to verify these preliminary findings.

## Key points

- Low-dose protocols allow the identification of relevant anatomical structures.
- Increasing the source-to-detector distance, the effective dose can be reduced by 59.5%.
- Cu filter addition of 0.1 mm can reduce the effective dose by 27.6%.
- Guidelines must be updated to concern digital technologies.

## Background

Lumbar spine radiography is a routine imaging examination performed to assess various conditions such as trauma, degenerative and neurologic symptoms [1]. The highest reported effective dose (ED) for this examination was 1.5 mSv [2–4], which is considered a high-radiation exposure, when compared to the average annual background radiation dose of 2 mSv received by the Australian population [5]. This high dose level is mainly related to the exposure settings, considering that examination is performed in one of the body areas that has the highest x-ray attenuation, thus requiring higher beam energy to penetrate the pelvic bones [3]. The imaging of this anatomical area also involves the exposure of radiosensitive reproductive organs [2, 6] and, for that reason, optimisation is critical since there is a potential risk of developing radiation-induced biological changes [2, 3, 6]. This is even more important in chronic conditions such as scoliosis and other spine congenital anomalies requiring repeated examinations.

Typically, the published literature that identified optimisation in radiography merely analysed the impact of a single-exposure parameter instead of taking into account all of them. The most widely studied exposure parameters identified in the literature were the source-to-detector distance (SDD) [7–9], tube voltage (kVp), tube current × exposure time product (mAs) [9, 10], additional beam filtrations [11–13], and type of projection (anterior-posterior, posterior-anterior, horizontal beam lateral) [2, 14–17]. These studies showed that a posterior-anterior lumbar spine radiogram was associated with a 65% ED reduction when compared to the anterior-posterior due to the attenuation of primary beam by the iliac bones [15, 16]. However, dose optimisation techniques for the routine lateral lumbar spine projection have not been fully explored in the current literature.

Additionally, the advent of digital radiography (DR) promoted optimisation opportunities due to the higher quantum efficiency of detector systems [11]. Considering that previous studies [8, 9, 18] assessed systems such as computed radiography (CR) or film-screen systems, it would be important to analyse the impact of the most recent technology, *i.e.*, DR [8, 9, 18].

Since the principle “As Low As Reasonably Achievable” (ALARA) must be applied in clinical context [2, 3, 8, 19], this study aims to address the research gap through the investigation of imaging parameters impact on lateral lumbar spine radiography reducing the ED whilst maintaining image quality (IQ) that allows the identifications of all relevant anatomical details.

## Methods

This study was performed in four phases: image acquisition, dose estimation, objective and perceptual IQ analysis. Ethical approval was obtained from Ethics Support Officer of Curtin University, Perth, Australia. Consent was obtained from participants to analyse image quality.

### Phase 1: image acquisition

**Fig. 1:**
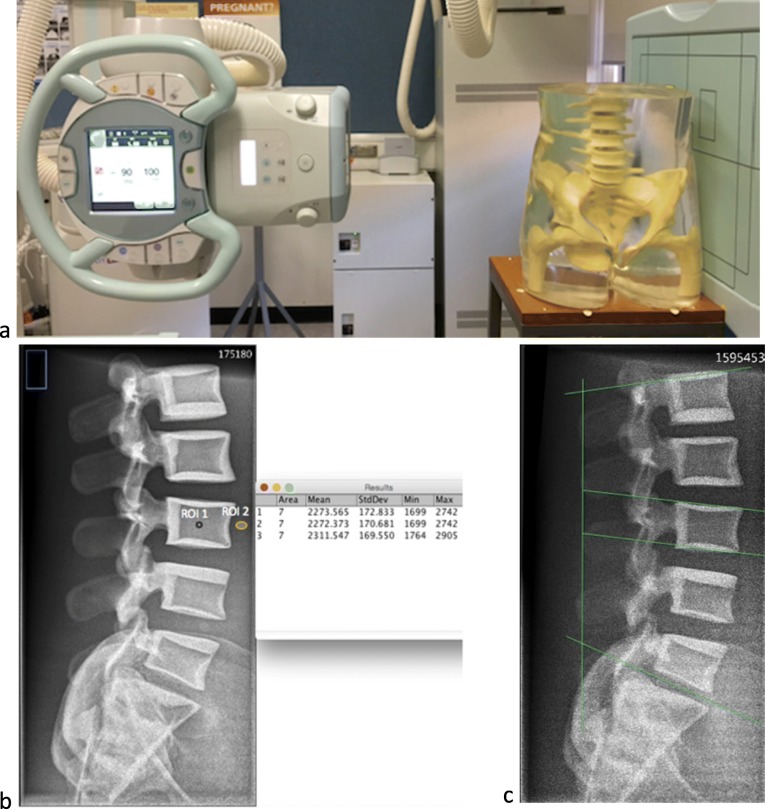
**a** Equipment setting for image acquisition. **b** Reference image acquired with baseline protocol and with contrast-to-noise ratio measurement using ImageJ software. **c** Straight lines drawn on the image with the lowest effective dose ED and contrast-to-noise ratio in the task for identification of relevant anatomical structures

An anthropomorphic phantom (STT/1163. Supertech, Inc., Elkhart, USA) that simulates a standard adult body habitus was used to produce lateral lumbar spine radiographs (Fig. 1a). The radiography unit used was a RAD speed general radiography unit (Shimadzu, Kyoto, Japan). The DR detector used for the image acquisition was a Canon CXDI-70C wireless caesium iodide flat panel display, with a pixel size of 125 μm and a matrix of 2800 × 3408.

The baseline protocol (Table 1) was obtained through a combination of multiple parameters proposed by the European Commission Guidelines [20], previous studies and data provided by clinical practice, being possible to obtain mean values. From here, subsequent manipulations were made to produce images with lower dose. The manipulated SDD ranged from 100 to 150 cm as proposed by European Commission guidelines, being selected the extremes and a middle value (130 cm) to verify if differences on IQ and dose were noticeable [3, 7, 8, 14, 20]. The tube potentials varied from 75, 85, and 95 kVp as suggested in multiple studies previously performed [2, 3, 7, 20, 21] and also by the European Commission guidelines [20]. The baseline mAs was determined at 18 mAs using the central sensor of the automated exposure control system and the subsequent values were achieved in accordance to the 10-kVp rule, which means that the mAs was halved with an increase of tube potential by 10 kVp [7, 10, 14]. Additional copper (Cu) filters with varying thicknesses of 0.1, 0.2, and 0.3 mm were used [3, 7, 12, 13, 22, 23]. All images were acquired with the same DR system, broad focus, constant collimation (16 × 23 cm) and stationary anti-scatter grid (ratio, 10:1; frequency, 52 lines cm^−1^, and focal distance, 100 cm) [7].

**Table 1:** Imaging parameters used to acquire 36 images (12 images per different source-to-detector distance (SDD)

Manipulated imaging parameters				Number of images
SDD (cm)	Beam energy (kVp)	Intensity (mAs)	Cu filter (mm)	
100, 130, or 150	75	18	0.0	12 × 3 = 36
	75	18	0.1	
	75	18	0.2	
	75	18	0.3	
	85	9	0.0	
	85	9	0.1	
	85	9	0.2	
	85	9	0.3	
	95	4.5	0.0	
	95	4.5	0.1	
	95	4.5	0.2	
	95	4.5	0.3	

### Phase 2: effective dose estimation

The ED was estimated using PCXMC 2.0 software based on Monte-Carlo simulations [2, 3, 7, 9, 10]. The estimation was performed by selecting ICRP 103 tissue weighting factors and using the exposure factors (kVp, mAs), SDD, focal-skin distance, collimation field and additional filtration parameters [2, 7, 10, 11] selected for each image acquisition.

### Phase 3: objective IQ assessment

During this phase, both contrast-to-noise ratio (CNR) and magnification were measured and calculated using ImageJ software version 2.0 (National Institutes of Health, Bethesda, MD, USA) [24]. In order to calculate CNR, regions of interest (ROIs) were applied on the vertebral body of L4 and on its adjacent homogenous background (Fig. 1b) using the following equation [7]:

$$ \mathrm{CNR}=\frac{\mathrm{Mean}\ \mathrm{pixel}\ \mathrm{value}\ \mathrm{ROI}\ 1-\mathrm{Mean}\ \mathrm{pixel}\ \mathrm{value}\ \mathrm{ROI}\ 2}{\mathrm{Standard}\ \mathrm{deviation}\ \mathrm{of}\ \mathrm{the}\ \mathrm{background}} $$

To determine the differences in magnification between images acquired at different SDDs, the area measurement function in ImageJ [2] was used at L3 vertebral body level and L5–S1 intervertebral disc space for all images. The magnification factor was then determined by dividing the measured area of a specific image by the measured area of reference image [25].

The selection of these specific locations for ROI measurements was based on the IQ criteria that are necessary to include in lateral lumbar spine radiography [20]. The vertebral bodies need to be clearly visualised to determine the spinal alignment and to detect any pathology such as fractures, lesions, deviations, degenerative process, or infections. Thee ROIs were defined at the L3 lumbar vertebral body because it is the central structure of this spine segment. To measure the contrast, it was then compared to the adjacent background [20, 23, 25].

### Phase 4: perceptual IQ assessment

Perceptual IQ assessment was performed to obtain the opinions expressed by independent human observers. Two tasks were included in this assessment, one dedicated to image scoring (Table 2) and another focused on drawing lines on specific anatomical details that are relevant when an image is assessed to verify if it meets the criteria considered as necessary to perform diagnosis. This assessment was completed by six observers with common radiography background (four fourth-year radiography students and two radiographers). Observers with a radiography background were selected, because in clinical situations they are the responsible professionals that decide whether or not to accept or reject the acquired images, by assessing its quality and to check if all criteria required are fulfilled to answer the clinical question. Two luminance levels of 170 and 25 lux were setup in the room to simulate possible variations observed in clinical practice. The x-ray rooms where radiographers assess the images are not typically setup to perform report and because of that, the lights on can have different levels of luminance.

**Table 2:** Image quality criteria and scoring scale applied to compare the reference image with the other acquired images

In comparison to the reference image		Scoring scale
1.	How would you rate the sharpness of the superior endplates of each lumbar vertebra on this radiograph?	-2 = much worse -1 = worse 0 = equal + 1 = better + 2 = much better
2.	How would you rate the sharpness of the inferior endplates of each lumbar vertebra on this radiograph?	
3.	How would you rate the outline of each intervertebral disc space on this radiograph?	
4.	Overall, how would you rate the amount of image noise on this radiograph?	
5.	Overall, how would you rate the image contrast of this radiograph?	
6.	Overall, how would you rate the image quality of this radiograph?	

During the assessment, two computer monitors in a computer lab were used. One of them constantly showed the reference image and the other monitor showed the other images to compare, one at a time. The monitors used were a 55-cm (1920 × 1080 pixels) BenQ GE2270-T light-emitting diode with anti-glare (BenQ Corporation, Neihu, Taipei, Taiwan). Both monitors were calibrated and assessed according to the recommendation of the American Association of Physicists in Medicine Task Group 18 through a series of visual assessments to ensure its suitability for the display of medical imaging. Uniformity was observed and no artefacts were identified [26].

For the image scoring purpose, images were compared to a reference image (acquired at 100 cm SDD, 75 kVp, 18 mAs, without additional Cu filter) according to the criteria presented in Table 2 and using a 5-point Likert scale [7].

To draw the lines on the relevant anatomical details, seven images were selected (one reference image and six images with the lowest ED) (Fig. 1c) and the task was performed using Radiant DICOM viewer (64-bit) (Poznan, Poland). The anatomical structures were chosen based on the proposed assessment criteria of lumbar spine radiography [1, 2, 7, 8, 20]. This task aimed to confirm each observer’s ability to identify the anatomical details on the images produced with lowest ED [7].

### Statistical analysis

The descriptive statistical analysis was conducted using the Statistical Package for the Social Sciences version 24.0 (IBM SPSS, Chicago, USA) and Excel 2017 (Microsoft Corporation, Redmond, WA, USA). A paired *t* test was performed to test the statistical significance between the results collected with two luminance levels. A *p* value less than 0.05 was used to verify statistical significance. The intraclass correlation coefficient (ICC) was used to report the level of agreement between and within the observers [7, 27, 28], interpreted as follows: < 0.4, poor reproducibility; 0.4–0.75, fair to good reproducibility and > 0.75, excellent reproducibility [7, 27]. The scoring scale of -2, -1, 0, 1 and 2 was adjusted to 1, 2, 3, 4 and 5 to facilitate the descriptive statistical analysis.

## Results

### Effective dose

**Fig. 2:**
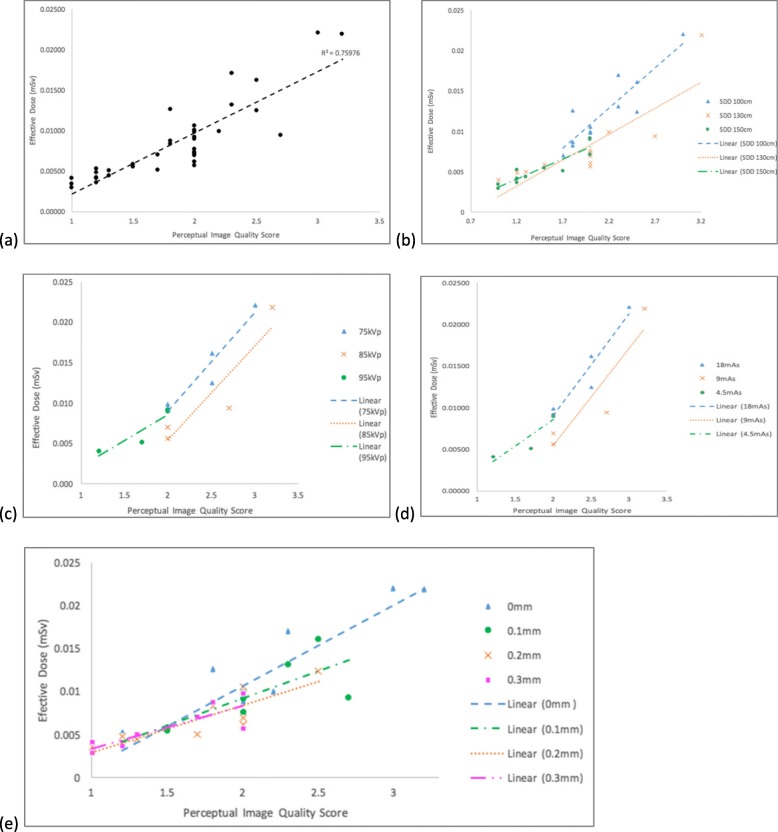
**a** Correlations between effective dose (ED) and perceptual image quality score. **b** Impact on ED and perceptual image quality score by changing source-to-detector distance (SDD), (**c**) kVp, (**d**) mAs, (**e**) Cu filter

The ED ranged from 0.003 to 0.022 mSv with the lowest values achieved using larger SDD (130 or 150 cm) in 24 out of 36 images and higher values were registered in images classified with larger scores (Fig. 2a). The reference image acquired at 100 cm SDD, 75 kVp, 18 mAs and without Cu filter had an ED of 0.022 mSv. Increasing the SDD to 150 cm whilst keeping the other imaging parameters constant resulted in 59.5% ED reduction (0.008 mSv) (Fig. 2b). Regarding the manipulation of beam energy and intensity, it was observed that ED was reduced by increasing the beam energy (Fig. 2c, d).

Comparing the ED values achieved in the images acquired without additional filters (0.012 mSv) to the images generated with additional Cu filter (0.007 mSv), a 43.7% ED reduction was noted. The ED was also reduced when the thickness of the additional Cu filter increased (Fig. 2e). The addition of 0.1-mm, 0.2-mm and 0.3-mm Cu filter resulted in 27.6% (0.009 mSv), 44.5% (0.006 mSv), and 42.3% (0.007 mSv) ED reduction, respectively.

There were six combinations of imaging parameters that generated the lowest ED values amongst all images (Table 3). All of the six images were generally acquired at larger SDD (130 or 150 cm), high tube voltage (85 or 95 kVp), low mAs (4.5 or 9 mAs) and with additional Cu filters. The image generated at the lowest ED was acquired with 150 cm, 95 kVp, 4.5 mAs, and 0.3-mm Cu filter (Fig. 1c).

**Table 3:** Imaging parameters: source-to-detector distance (SDD), beam nergy (kVp), beam intensity (mAs), additional copper filtration (Cu filter), contrast-to-noise ratio (CNR), mean perceptual image quality (IQ) score, effective dose (ED), and change in ED in comparison with the reference image, highest IQ score image, and sixth lowest ED images (14)

Image	SDD	kVp	mAs	Additional Cu filter (mm Al)	CNR	Mean IQ score ± SD	ED (mSv)	Change in ED (%)
Highest IQ score	130	75	18.0	0.0	7.23	0.6 ± 0.41	0.0218	-0.70
Highest ED (Ref. image)	100	75	18.0	0.0	7.18	0.0 ± 0.0	0.0220	0.00
6th lowest ED	150	95	4.5	0.1	2.99	-1.7 ± 0.41	0.0042	-80.90
5th lowest ED	150	75	18.0	0.3	3.5	-1.8 ± 0.41	0.0041	-81.50
4th lowest ED	130	95	4.5	0.3	2.24	-1.8 ± 0.41	0.0040	-81.60
3rd lowest ED	150	85	9.0	0.3	3.22	-1.8 ± 0.41	0.0036	-83.60
2nd lowest ED	150	95	4.5	0.2	2.56	-2.0 ± 0.0	0.0035	-85.0
Lowest ED (and IQ score)	150	95	4.5	0.3	2.13	-2.0 ± 0.0	0.0029	-87.0

*SD* Standard deviation

### Image quality: CNR and magnification

The calculated CNR values ranged from 2.13 to 7.23 (Table 3). The images acquired at larger SDDs (130 or 150 cm) were characterised by a lower CNR (4.22 and 3.71) when compared to those produced with 100-cm SDD (4.51). The highest overall CNR value was achieved at the lowest 75 kVp and the highest 18 mAs. The application of additional Cu filter also impacted the CNR as it decreased from 5.18 to 3.24 with the increase on Cu filter thickness from 0 to 0.3 mm.

Amongst the six images performed at lower ED and CNR values, the 3^rd^ lowest ED value was associated with an image with a CNR of 3.22, which was higher than the one with the 6^th^ lowest ED (Table 3). The image with the highest CNR was acquired with an ED lowered by 83.6% (0.003 mSv) when compared to the reference image (0.022 mSv). The imaging parameters of the 3^rd^ lowest ED image were 150 cm, 85 kVp, 9 mAs, and 0.3-mm Cu filter. Magnification reduction was observed at larger SDD having factors of 0.91 and 0.86 for 130-cm and 150-cm SDD, respectively.

### Image quality: observer assessment

There was no significant difference between the results collected at the two different luminance levels (*p* = 0.491). The provided perceptual IQ scores varied between -2 and 1. Results showed that only one image was rated better than the reference image whilst the other 35 images were rated as worse or much worse score. The highest quality score (1) was attributed to the image acquired at 130 cm, 75 kVp, 18 mAs, and without Cu filter. For the six images produced with the lowest ED (Table 3), observers commonly scored them with the values varying between -1.7 and -2.

The inter- and intra-observer ICC varied from moderate to excellent, with the first presenting a range between 0.61 and 0.79, whilst the second ranged from 0.55 to 0.82.

Although the six images used in the line drawing task were associated with the lowest ED and CNR, all observers were able to competently identify the relevant anatomical structures as demonstrated by drawing in the images straight lines in the required anatomical structures (Fig. 1c).

## Discussion

Several studies have investigated the x-ray dose optimisation techniques of lumbar spine radiography. However, the majority focused on the manipulation of a single imaging parameter instead of considering the imaging parameters as combinations [2, 3, 7, 9, 14–16, 25]. In this study, the baseline protocol was determined by considering the different imaging parameters proposed by the current literature. Investigations were then conducted by manipulating the baseline protocol to identify the optimal combinations to achieve an ED reduction whilst maintaining an IQ allowing the identification of relevant anatomical structures.

The ED could be reduced when compared to the suggestions promoted by the European Guidelines for lateral lumbar spine [20] and also by previous studies through manipulations of several exposure parameters and utilisation of different techniques, such as using larger SDD. This is an expected outcome as the inverse square law states that the radiation intensity is inversely proportional to the radiation source distance [8, 14, 18]. In alignment with previous findings, this study also indicates the efficacy of applying the 10-kVp rule in reducing ED when manipulating kVp and mAs [7, 9, 10, 14]. This is because increasing the kVp itself would increase the dose, but with a concomitant decrease of mAs, would reduce the resulting ED [7, 9, 29]. Another technique for ED reduction is the use of additional Cu filter. As evident in this study, as well as in previous studies [7, 10, 11, 23], the selection of the higher thickness of Cu filter could produce lower ED values. With additional beam filtration, the low-energy spectrum of x-rays will be removed, which consequently increases the penetration energy reducing the radiation absorption by the body tissues [7, 12, 13, 22, 23].

Whilst ED reduction was observed, the IQ was decreased accordingly. Using the larger SDD (130 or 150 cm) had resulted in lower CNR values compared to that of 100-cm SDD. This is attributed by the beam divergence that reduces the radiation intensity, which subsequently deteriorates the IQ or lowers the CNR [7, 14, 18]. Another factor that leads to a lower CNR is the selection of higher kVp values due to the increased amount of scattered radiation that would impinge on the IQ with higher noise [7, 29, 30]. The application of additional Cu filter also resulted in a lower CNR due to the beam hardening effect that consequently decreases the associated ED and IQ [7, 13, 22].

Another IQ aspect that was affected by SDD was the magnification. It was observed that the magnification decreased when the SDD increased. This outcome is expected since the ratio between SDD and source-to-object distance is higher, whilst the object-to-image receptor distance remains constant despite using different SDD [31].

In the perceptual IQ assessment, there was no significant difference between the results collected with different luminance levels. The good level of agreement could be contributed by the similar medical imaging backgrounds and knowledge amongst the recruited observers in this study. Nevertheless, the lighting conditions should be taken into considerations as it was reported that different luminance levels could have impacts on the observers’ perceptions during radiographic image analysis [32].

As is expected from this study, the perceptual IQ scores increased along with the ED [2, 8, 11, 29]. For instance, the highest CNR (7.23) was associated with 0.022 mSv whereas the lowest CNR (2.13) was obtained at the lowest ED (0.003 mSv) (Table 3). This comparison confirms that the generation of higher IQ can only be achieved at the cost of higher ED [7, 29]. For the observers, the images produced with lower ED (< 0.009 mSv) had the same low score, potentially justified by the higher level of noise, which to the human’s eye can be perceived as similar. However, a lower IQ still allowed the identification of all relevant anatomical structures. Although the low-dose protocols generated the images with suboptimal quality, all observers were still able to draw straight lines across the specified anatomical structures relevant for this examination. Therefore, results of this study further prove the possibility of using low-dose protocols whilst maintaining an IQ that allows image analysis regarding anatomy, although further studies are necessary to verify the impact on pathology identification [7, 14, 22, 23, 29]. Upon analysing the results, the ED reduction in lateral lumbar spine radiography can be performed by applying larger SDD (130–150 cm), higher kVp (85–95 kVp), lower mAs (4.5–9 mAs), and additional Cu filter (0.1–0.3 mm).

The main limitation of this study is the utilisation of the anthropomorphic phantom that only simulates the radiation absorption properties of a standard adult body habitus. Hence, future research should include real patient data whilst taking into account the different types of body habitus within clinical practice, namely the wide ranges from paediatric to adult obese patients. Another limitation is the potential observer bias during the perceptual IQ assessment. This is because the recruited observers had prior knowledge about their performing tasks before the commencements, which could potentially affect their responses [33]. Thus, future research could minimise this bias by implementing clear rules and procedures for a task whilst specifying a time limit for its completion. The third limitation is the location for the perceptual IQ assessment, which was performed in a computer lab. Considering the conditions of this location, it may not fully simulate the working conditions in the radiography practice used for image analysis to meet the image criteria required for each context. The absence of pathology in assessment is another limitation. This is because in the majority of contexts, not only the anatomy is assessed but also the low contrast lesions that should be visible on the images. This could be addressed by reviewing the patient’s images with different pathological lesions and complete the study using receiver operating characteristic analysis to verify the limits of the system to detect pathology. The spatial resolution can also be assessed in the future. This study did not include this measurement because the main variables tested were beam energy and intensity and that does not impact on it. The SDD can affect spatial resolution but considering the magnification factor achieved varied between 1 and 0.86 it was decided to neglect.

In conclusion, this study showed that application of larger SDD (130 or 150 cm), higher tube voltage kVp (85 or 95 kVp) with lower mAs (4.5 or 9 mAs) and additional Cu filter (0.1, 0.2, or 0.3 mm) can reduce the ED by 63% compared to the protocols proposed by the literature. The lowest ED and IQ were acquired using the imaging parameters of 150 cm SDD, 95 kVp, 4.5 mAs, and additional 0.3-mm Cu filter. Although the images were found to be associated with the lowest CNR (2.13) and lowest IQ score (-2), the IQ was still considered acceptable as it allows all observers to identify relevant anatomical structures. Future research should consider analysing the real patient data, including different body habitus, pathologies, receiver operating characteristic analysis and applying real clinical conditions for a more realistic assessment.

## Data Availability

Data generated or analysed during this study are included in this published article.

## Abbreviations

CNR: Contrast-to-noise ratio
DR: Digital radiography
ED: Effective dose
IQ: Image quality
ROI: Region of interest
SDD: Source-to-detector distance
